# Associations of green tea, coffee, and soft drink consumption with longitudinal changes in leukocyte telomere length

**DOI:** 10.1038/s41598-022-26186-y

**Published:** 2023-01-10

**Authors:** Inhae Sohn, Chol Shin, Inkyung Baik

**Affiliations:** 1 Department of Foods and Nutrition, College of Science and Technology, Kookmin University, 77 Jeongneung-ro, Songbuk-gu, Seoul, 02707 Korea; 2 Department of Internal Medicine, Korea University Ansan Hospital, 123 Jeokgeum-ro, Danwon-gu, Ansan, 15355 Korea

**Keywords:** Biomarkers, Risk factors

## Abstract

Whether beverage consumption is associated with longitudinal observation of telomere length remains unclear. We evaluated the association of green tea, coffee, and soft drink consumption with 6-year changes in leukocyte telomere length (LTL). The study included 1952 participants who provided whole blood samples for LTL assays during the baseline (year 2011–2012) and follow-up (year 2017–2018) periods and reported baseline information on consumption of green tea, coffee, and soft drinks. Robust regression analysis was used to analyze the association adjusted for potential confounding variables. In the results, an inverse association between green tea consumption and LTL changes from baseline, which indicate telomere shortening, was found; regression coefficient [95% confidence interval] was − 0.097 [− 0.164, − 0.029] for participants who daily consumed at least 1 cup of green tea compared with non-consumers (*p* value = 0.006). This association was stronger among women (versus men) and younger participants aged 50–64 years (versus older). However, a positive association between soft drink consumption and LTL shortening was observed among women (*p* value < 0.05). Coffee consumption was not associated with LTL changes. These findings suggested that green tea consumption may be protective against telomere shortening reflecting biological aging whereas coffee and soft drink consumption may not.

## Introduction

Telomeres consisting of repeated DNA sequences (TTAGGG) are known to play a role in protecting the ends of human chromosomes from fusions and degradation. As cells replicate, telomere length shortens due to the end-replication problem leading to cellular senescence^1,2^. Recently, many epidemiological studies measured leukocyte telomere length (LTL) as a useful marker of biological aging and accumulating data showed an inverse association between LTL and chronological age^3^. Some studies have provided data on the association of LTL with obesity, diabetes mellitus, hypertension, cardiovascular diseases, dementia, and cancer, including all-cause mortality^4–9^.

It was reported that LTL is influenced by genetic and lifestyle factors, including diet and alcohol consumption^10–13^. A few studies showed a significant association of LTL with the consumption of beverages, such as tea^14^, coffee^15^, and sweetened beverages^12,16^. These associations were analyzed in cross-sectional data while longitudinal data regarding changes in LTL are still limited. There is one intervention study which has observed LTL changes with beverage consumption as an exposure and found increased LTL in obese women with two-month green tea supplementation^17^. Although this study used a high dose of supplementation equivalent to at least 10 cups/day of green tea for a short period, it provides primary data to support the association between green tea consumption and longitudinal changes in LTL. Based on previous epidemiological studies reporting the significant associations of mortality with green tea and coffee consumption^18^ as well as with LTL changes^19,20^, it is worthy to investigate the link between these beverages and LTL changes in terms of exploring implications for lifestyle factors related to longevity.

The present study, which was embedded in a population-based cohort study, conducted repeated assays of LTL and aimed to investigate the association of green tea, coffee, and soft drink consumption with cross-sectional LTL and longitudinal changes in LTL during a six-year period among middle-aged and older Korean men and women. Furthermore, it evaluated whether the association results are different according to age groups and sex.

## Results

Characteristics, including beverage consumption status of the study participants, were compared across the tertile groups of LTL changes (Table 1). According to higher tertiles indicating greater LTL shortening, baseline values were greater whereas follow-up values of LTL were less. Participants with greater LTL shortening were more likely to be males and current smokers and consumed less green tea (*p *value for trend < 0.05). Compared with participants in the first and second tertiles, those in the top tertile tended to have less consumption of green tea and brewed coffee but consumed greater amount of instant coffee and soft drinks. Those in the second tertile showed greatest consumption of other types of tea.

**Table 1:** Characteristics of the 1952 participants according to tertile groups of six-year changes in leukocyte telomere length.

Variables	All participants		Tertile of changes in leukocyte telomere length			*p* value for trend
	[median]		1st tertile	2nd tertile	3rd tertile	
Number of participants (%)	1952 (100)		650 (33.3)	651 (33.4)	651 (33.4)	
**Baseline and follow-up LTL data**						
LTL changes^1^	− 0.03 ± 0.54	[− 0.02]	− 0.58 ± 0.37	− 0.02 ± 0.10	0.52 ± 0.38	< 0.001
Baseline LTL	1.08 ± 0.40	[0.98]	0.86 ± 0.25	0.97 ± 0.22	1.40 ± 0.46	< 0.001
Follow-up LTL	1.10 ± 0.40	[1.02]	1.44 ± 0.43	0.99 ± 0.23	0.88 ± 0.25	< 0.001
**Baseline data of characteristics**						
Age, years	58.1 ± 6.9	[56.0]	58.4 ± 7.0	58.0 ± 6.8	57.8 ± 6.7	0.12
Men, %	50.0		46.3	50.5	53.2	< 0.05
Low income^2^, %	17.0		18.9	16.3	15.9	0.16
Employed, %	34.9		30.9	37.2	36.6	< 0.05
Body mass index, kg/m^2^	24.7 ± 3.0	[24.6]	24.7 ± 3.0	24.7 ± 2.9	24.8 ± 3.0	0.80
Current smoker, %	12.5		9.9	13.5	14.0	0.02
Current alcohol drinker, %	48.7		45.4	50.2	50.5	0.06
Physical activity, met-h/day	40.9 ± 6.3	[40.0]	40.9 ± 5.9	40.7 ± 6.4	41.1 ± 6.6	0.65
Presence of hypertension, %	34.1		33.7	33.5	35.2	0.57
Presence of diabetes mellitus, %	18.1		17.9	18.0	18.4	0.78
White blood cell counts, thousand/uL	5.1 ± 1.5	[4.9]	5.0 ± 1.5	5.1 ± 1.4	5.2 ± 1.5	0.06
**Beverage consumption, cup/week**						
Green tea	1.8 ± 6.2	[0]	2.1 ± 6.2	2.0 ± 7.4	1.4 ± 4.5	0.04
Other types of tea^3^	0.27 ± 2.81	[0]	0.19 ± 1.61	0.49 ± 4.34	0.14 ± 1.51	0.77
Brewed coffee	2.5 ± 7.1	[0]	2.5 ± 7.0	3.0 ± 7.9	2.1 ± 6.2	0.38
Instant coffee	8.9 ± 11.6	[7]	8.6 ± 11.5	8.9 ± 11.5	9.4 ± 11.9	0.80
Soft drink	0.17 ± 1.46	[0]	0.16 ± 1.75	0.13 ± 0.86	0.20 ± 1.60	0.61

Values are mean ± standard deviation or %.

^1^Changes were calculated by subtracting the follow-up value from the baseline value of telomere length (baseline value − follow-up value) and its positive value indicates telomere shortening.

^2^Monthly household income less than 1,500,000 won (approximately equal to 1300 dollars).

^3^Black tea, oolong tea, and other teas.

### Cross-sectional and longitudinal associations between beverage consumption and LTL

A supplemental table shows results regarding the association between beverage consumption and cross-sectional observations for LTL (Supplemental Table S1). No significant association between beverage consumption and baseline LTL was observed. In the multiple model for follow-up LTL, participants with green tea consumption ≥ 7 cups/week showed longer LTL values compared with those who did not drink green tea (*p* < 0.05).

Table 2 presents regression coefficient estimates and their 95% confidence intervals (95% CI) for the association between beverage consumption and longitudinal observations for LTL. As shown, green tea drinkers showed less LTL shortening during the six-year period compared with nondrinker (*p* < 0.01). After further adjustment of the baseline LTL in the multiple model, this significant association was not changed (data available upon request). No significant association was observed for other beverages.

**Table 2:** Association between beverage consumption and 6-year changes^1^ in leukocyte telomere length.

Beverage consumption	Categories	N^2^	Age, sex, WBCC adjusted model	Multiple model^4^
Types			Estimate^3^ (95% CI)	Estimate^3^ (95% CI)
Green tea	None	1505	Reference	Reference
	< 7 cups/week	227	− 0.024 (− 0.090, 0.043)	− 0.021 (− 0.089, 0.044)
	≥ 7 cups/week	220	− 0.094 (− 0.161, − 0.027)^6^	− 0.097 (− 0.164, − 0.029)^6^
Others^5^	None	1910	Reference	Reference
	≥ 1 cup/month	42	− 0.054 (− 0.199, 0.090)	− 0.052 (− 0.197, 0.093)
Brewed coffee	None	1563	Reference	Reference
	< 14 cups/week	217	0.035 (− 0.033, 0.103)	0.037 (− 0.031, 0.105)
	≥ 14 cups/week	172	− 0.030 (− 0.106, 0.045)	− 0.030 (− 0.105, 0.046)
Instant coffee	None	755	Reference	Reference
	< 14 cups/week	518	− 0.001 (− 0.054, 0.052)	− 0.001 (− 0.054, 0.053)
	≥ 14 cups/week	679	− 0.009 (− 0.061, 0.043)	− 0.012 (− 0.064, 0.041)
Soft drink	None	1870	Reference	Reference
	≥ 1 cup/month	82	− 0.009 (− 0.115, 0.096)	− 0.008 (− 0.113, 0.098)

*CI* confidence interval, *WBCC* white blood cell counts.

^1^Changes were calculated by subtracting the follow-up value from the baseline value of telomere length (baseline value − follow-up value) and its positive value indicates telomere shortening.

^2^Number of participants.

^3^Regression coefficient estimate.

^4^Model adjusted for age, sex, monthly household income status, employment status, body mass index, smoking status, alcohol consumption status, physical activity, white blood cell counts, and presence of hypertension or diabetes mellitus.

^5^Black tea, oolong tea, and other types of tea.

^6^*p* value = 0.006.

### Stratified analyses by age groups and sex for the longitudinal association between beverage consumption and LTL

Results of the association between beverage consumption and LTL changes stratified by age groups are shown in Table 3. As shown, a significant inverse association between green tea consumption ≥ 7 cups/week and LTL shortening was solely observed among participants younger than 65 years (*p* < 0.01). However, no significant association was observed among older participants. Because this null association might be partly due to a small number of those with 65 years or older, we re-analyzed data using 60 years as a cutoff point of age and observed similar results (data available upon request). Other types of beverages consumed were not associated with LTL shortening.

**Table 3:** Age-stratified association between beverage consumption and six-year changes^1^ in leukocyte telomere length.

Beverage consumption	Categories	Model^3^ for < 65 years		Model^3^ for ≥ 65 years	
Types		N^2^	Estimate^4^ (95% CI)	N^2^	Estimate^4^ (95% CI)
		1578		374	
Green tea	None	1198	Reference	307	Reference
	< 7 cups/week	189	− 0.003 (− 0.075, 0.070)	38	− 0.118 (− 0.292, 0.055)
	≥ 7 cups/week	191	− 0.114 (− 0.185, − 0.042)^6^	29	0.052 (− 0.147, 0.252)
Others^5^	None	1542	Reference	368	Reference
	≥ 1 cup/month	36	− 0.112 (− 0.268, 0.043)	6	0.154 (− 0.263, 0.571)
Brewed coffee	None	1222	Reference	341	Reference
	< 14 cups/week	197	0.031 (− 0.041, 0.102)	20	0.167 (− 0.069, 0.403)
	≥ 14 cups/week	159	− 0.042 (− 0.120, 0.036)	13	0.047 (− 0.242, 0.336)
Instant coffee	None	586	Reference	169	Reference
	< 14 cups/week	414	0.007 (− 0.053, 0.066)	104	− 0.039 (− 0.165, 0.088)
	≥ 14 cups/week	578	− 0.009 (− 0.066, 0.048)	101	− 0.034 (− 0.172, 0.104)
Soft drink	None	1504	Reference	366	Reference
	≥ 1 cup/month	74	− 0.013 (− 0.123, 0.097)	8	− 0.153 (− 0.531, 0.224)

CI, confidence interval.

^1^Changes were calculated by subtracting the follow-up value from the baseline value of telomere length (baseline value − follow-up value) and its positive value indicates telomere shortening.

^2^Number of participants.

^3^Model adjusted for sex, monthly household income status, employment status, body mass index, smoking status, alcohol consumption status, physical activity, white blood cell counts, and presence of hypertension or diabetes mellitus.

^4^Regression coefficient estimate.

^5^Black tea, oolong tea, and other teas.

^6^*p* value = 0.002.

Table 4 presents results stratified by gender for the association between beverage consumption and LTL changes. A significant inverse association between green tea consumption ≥ 7 cups/week and LTL shortening was shown among women only after adjusting for potential confounding variables (*p* < 0.05). Although no association for soft drinks was observed among all participants in Table 2, a significant positive association was observed among women (*p* < 0.05). In contrast, an insignificant inverse trend was examined among men. For other beverages, no significant association was observed.

**Table 4:** Sex-stratified association between beverage consumption and six-year changes^1^ in leukocyte telomere length.

Beverage consumption		Model^3^ for women		Model^3^ for men	
Types	Categories	N^2^	Estimate^4^ (95% CI)	N^2^	Estimate^4^ (95% CI)
		976		976	
Green tea	None	737	Reference	768	Reference
	< 7 cups/week	140	− 0.088 (− 0.176, 0.001)	87	0.075 (− 0.028, 0.178)
	≥ 7 cups/week	99	− 0.104 (− 0.206, − 0.001)^6^	121	− 0.083 (− 0.173, 0.006)
Others^5^	None	953	Reference	957	Reference
	≥ 1 cup/month	23	− 0.027 (− 0.228, 0.175)	19	− 0.098 (− 0.309, 0.114)
Brewed coffee	None	738	Reference	825	Reference
	< 14 cups/week	142	0.037 (− 0.052, 0.126)	75	0.044 (− 0.067, 0.154)
	≥ 14 cups/week	96	− 0.029 (− 0.135, 0.076)	76	− 0.034 (− 0.144, 0.076)
Instant coffee	None	476	Reference	279	Reference
	< 14 cups/week	311	− 0.010 (− 0.080, 0.060)	207	0.014 (− 0.070, 0.098)
	≥ 14 cups/week	189	− 0.021 (− 0.104, 0.063)	490	− 0.009 (− 0.079, 0.060)
Soft drink	None	953	Reference	917	Reference
	≥ 1 cup/month	23	0.230 (0.030, 0.431)^6^	59	− 0.095 (− 0.217, 0.027)

*CI* confidence interval.

^1^Changes were calculated by subtracting the follow-up value from the baseline value of telomere length (baseline value − follow-up value) and its positive value indicates telomere shortening.

^2^Number of participants.

^3^Model for leukocyte telomere length adjusted for age, monthly household income status, employment status, body mass index, smoking status, alcohol consumption status, physical activity, white blood cell counts, and presence of hypertension or diabetes mellitus.

^4^Regression coefficient estimate.

^5^Black tea, oolong tea, and other teas.

^6^*p* value < 0.05.

## Discussion

In this study, we evaluated six-year changes in LTL among 1952 Korean adults and its association with beverage consumption, including tea, coffee, and soft drinks. In our findings, daily consumption of green tea was inversely associated with LTL shortening, a change from baseline to follow-up values, whereas soft drink consumption was positively associated with it. In particular, these associations were more evident among women than among men. This association for green tea consumption was also significant among participants younger than 65 years. Additionally, consumption of coffee and other types of tea was associated with neither cross-sectional values nor longitudinal changes of LTL.

An earlier epidemiological study reported a positive association between tea consumption and cross-sectional values of LTL^14^. Although further data for the association between varied types of tea and LTL are limited, the beneficial effects of green tea, oolong, and black tea, which are favorably consumed worldwide, have been well described^21–23^. These teas contain polyphenols, tocopherols, ascorbic acid, carotenoids, phytochemical compounds, and minerals such as Cr, Mn, Se, or Zn. Specifically, green tea contains catechins, which are polyphenolic compounds classified as flavonoids, showing robust antioxidant properties^24^, gallic acid, and other phenolic acids^25^. Polyphenol epigallocatechin-3-gallate (EGCG), one of the most abundant catechins found in green tea, is a component with antioxidant and anti-inflammatory properties^26,27^. Furthermore, compounds with antioxidant properties scavenge reactive oxygen species (ROS) and nitrogen species and chelate redox-active metal ions, which are involved in Fenton and Haber–Weiss reactions. These scavenging properties are essential because ROS attacks the G triplets in telomeres, leading to DNA cleavage and telomere attrition^28–30^. Thus, antioxidant properties of green tea may delay telomere attrition by protecting telomeres against oxidative damage. As another mechanism, the impact of green tea compounds on telomerase may contribute to the maintenance or elongation of telomeres. According to an in vitro study, EGCG increased chromosomal instability (CIN) in a dose-dependent manner, reportedly correlated with telomere shortening, in colon adenocarcinoma cells, but it decreased CIN in normal colon epithelial cells, proposing different effects of EGCG on normal and malignant cells^31^. In normal cells, EGCG may inhibit telomere shortening by modifying the mechanism of telomere biology, for example, by upregulating telomerase activity. An intervention study demonstrated that obese women with green tea supplementation had increased LTL, possibly reflecting telomere lengthening^17^. Our study found a significant association between green tea consumption and LTL changes, in particular LTL lengthening, among women only. Biological mechanisms underlying the differential associations by sex are not fully understood. It has been suggested that men have faster attrition of telomere length than women partly due to the association of sex hormones in telomere maintenance and oxidative stress^32^, and thus there may be sex-specific effects of green tea on this association.

Some investigations reported conflicting data regarding the association between coffee consumption and LTL^11,12,33^. For example, a cross-sectional study showed no association^11^, while others observed a positive association^12,33^. The present longitudinal study observed a significant association between soft drink consumption and LTL shortening in women. An earlier animal data^34^ indicating that soft drink consumption induces oxidative stress may explain a biological mechanism underlying this association.

Study limitations should be considered when interpreting our findings. Telomere length was assessed in leukocytes rather than somatic cells in this study. Because it was reported that telomere shortening rates were similar in leukocytes and somatic cells^35^, LTL has been a valuable marker of biological aging. However, based on longitudinal changes in LTL, telomere lengthening has been proposed to be limited to hematopoietic cells^36^. We used the baseline information of beverage consumption as an exposure in this study because its follow-up information was unavailable. Under the assumption that the baseline information of beverage consumption is sustained during the study period, we analyzed its association with longitudinal changes in telomere length. Thus, the exposure of beverage consumption might be less accurate probably due to non-differential misclassification leading to weak or null associations. Thus, further investigation on coffee consumption and other beverages, except green tea, is warranted. In addition, unadjusted residual confounding factors including diet and dietary supplementations, which we were unable to observe in this study, might lead to weak or null associations.

The study findings can also be generalized to Korean adults of similar ages. Nevertheless, further studies including participants with varied ethnicities and age ranges are warranted. The strength of our study includes using a large sample size from the general population, a wide range of confounding variables, and the longitudinal observation method of LTL adopted.

Therefore, based on our findings, we suggest beneficial effects of green tea consumption and potentially disadvantageous effects of soft drink consumption on LTL shortening, which may reflect accelerated biological aging.

## Methods

### Study design and participants

Study participants were from an ongoing population-based prospective cohort study, a part of the Korean Genome Epidemiology Study. Detailed information on the study design and procedures is available in a previous study^37,38^. Briefly, eligible study subjects were selected based on a two-stage cluster sampling method using the information of residential district and demographic characteristics and identified by telephone contact. They were invited to visit the Korea University Ansan Hospital on a designated date between June, 2001 and January, 2003. Finally, 5012 participants were enrolled at baseline and have been followed up biennially since February, 2003. At baseline, trained researchers conducted a questionnaire-based interview and comprehensive health examination, including anthropometric assessment, blood pressure (BP) measurement, and bio-specimen collection. The questionnaire inquired about participants’ demography, medical history, health conditions, and lifestyle including smoking and alcohol drinking, and dietary intake. During the follow-up period, similar interviews and health examinations were repeated although food frequency questionnaire was not included after 2011. The Human Subjects Review Committee at the Korea University Ansan Hospital approved all study procedures and protocols, and participants signed an informed consent form during every visit (IRB number: ED0624). All methods were performed in accordance with the Declaration of Helsinki and local guidelines and regulations concerning the ethics of human research.

For the present study, initial investigation on beverage consumption, as an exposure, and LTL, as an outcome, was conducted between February, 2011 and November, 2012 (the baseline period). Then, repeated measurement of LTL was conducted between February, 2017 and December, 2018 (the follow-up period). Thus, longitudinal changes during six years between initial assays and repeated assays of LTL were observed. As recorded, 2314 cohort members completed the interview and health examination and their initial LTL were assayed. Among 84% of them, the follow-up assay of LTL were completed. Thus, 1952 participants were included in the analysis. General characteristics, except age, between participants and nonparticipants were similar.

### Beverage consumption

Information on green tea, coffee, and soft drink consumption was collected during the baseline period using a caffeine food frequency questionnaire (C-FFQ), but it was not updated during the follow-up period. C-FFQ inquired consumption of 18 beverage categories, including brewed coffee, three types of instant coffee, green tea, black tea, oolong, two types of instant hot chocolate, two types of regular coke, diet coke, two types of carbonated drinks, two types of energy drink, and two types of processed milk with the information on quantified consumption frequency (one standard serving size for beverage is defined as one paper cup of 190 mL) consumed over the past year. This questionnaire was constructed based on the Fred Hutchinson Cancer Research Center Caffeine Questionnaire^39^. We calculated average consumption per week for each beverage and generated categories considering the number of participants in each category; none, 0–14 cups/week, and ≥ 14 cups/week for brewed and instant coffee; none, 1–6 cups/week, and ≥ 7 cups/week for green tea; then none and ≥ 1 cup/week for other types of tea or soft drinks.

### Measurement of leukocyte telomere length

Relative LTL was measured using quantitative real-time polymerase chain reaction^40^. Genomic DNA in leukocytes was extracted from peripheral blood samples collected during the baseline and six-year follow-up periods, using a QIAamp DNA blood mini kit (Qiagen, Hilden, Germany). Then, purified DNA samples were diluted and quantified using a NanoDrop 1000 spectrophotometer (Thermo Fisher Scientific, Wilmington, DE, USA). Subsequently, the ratio of telomere repeat copy number to the single-copy gene copy number (36B4 gene encoding acidic ribosomal phosphoprotein) was determined using the iQ Multi-Color Real-Time PCR Detection System (Bio-Rad, Hercules, CA, USA). The final concentrations of the PCR reagents were 1× SYBR Green SuperMix (Bio-Rad), 50 ng of DNA, 0.2 μM of telomere primers (forward, 5′-GGTTTTTGAGGGTGAGGGTGAGGGTGAGGGTG A GGGT-3′; reverse, 5′-TCCCGACTATCCCTATCCC TATC CCTATCCCTATCCCTA-3′) and 0.3 μM of 36B4 primers (forward, 5′-CAGCAAGTGGGAAGGTGTAATC C-3′; reverse, 5′-CCCATTCTATCATCAACGGGTACAA-3′). Reactions were conducted using telomere and 36B4 primers in the same 96-well plate, and each plate included a reference DNA sample. Finally, a four-point standard curve was established to transform the cycle threshold to nanograms of DNA. In a quality control test, coefficient of variation values were 4.4% for the intra-assay and 7.6% for the inter-assay when 34 duplicate samples were run in two batches. To evaluate reproducibility, we obtained Pearson correlation coefficients; 0.97 (*p* < 0.001) for 34 duplicate samples, 0.98 (*p* < 0.001) for 17 duplicate samples in one batch, and 0.95 (*p* < 0.001) for 17 duplicate samples in the other batch. To calculate six-year changes in LTL, a change from baseline to follow-up values indicating LTL shortening for each individual was calculated.

### Potential confounding variables

On the basis of previous studies related to main exposures and outcomes^12,14^, we considered age, sex, body mass index (BMI), income status, smoking status, alcohol consumption status, physical activity, and presence of diabetes mellitus or hypertension as potential confounding variables. In this study, the baseline information on these confounding variables was used. Body weight and height were measured to the nearest 0.1 kg and 0.1 cm, respectively, to calculate BMI (kg/m^2^). Smoking status was categorized into four groups; nonsmoker and current smoker (≤ 10 cigarettes/day, 11–20 cigarettes/day, and > 20 cigarettes/day). Alcohol consumption status was also categorized into four groups; nondrinker and current drinker (< 15 g/day, 15–30 g/day, and > 30 g/day). To assess physical activity, participants were asked to report hours spent in a typical day in sleep and five categories of activity intensity (sedentary, very light, light, moderate, vigorous), for each of which specific activities were listed. A total metabolic equivalent (MET) score was calculated by multiplying hours spent by a MET value (1.0 for sleep or sedentary, 1.5 for very light, 2.4 for light, 5.0 for moderate, and 7.5 for vigorous activity), which was an average of MET values of specific activities listed for each category^41^, and summing up the total. The presence of diabetes mellitus or hypertension was determined if one of the following criteria was met: use of hypoglycemic medications or fasting plasma glucose levels ≥ 126 mg/dL or postprandial glucose level ≥ 200 mg/dL; use of hypertension treatment medications or systolic BP ≥ 140 mmHg or diastolic BP ≥ 90 mmHg.

### Statistical analysis

Descriptive statistics, chi-square test, and analysis of variance (ANOVA) were used to compare participants’ characteristics according to the categories of LTL changes. The Cochran-Armitage trend test and linear trend analysis in ANOVA were performed to obtain *p* values for trend. To analyze the association between beverage consumption and LTL changes, a robust linear regression model was used because the distribution of the dependent variable, which had negative and positive values and zero, was skewed due to outliers. In multiple models, age, BMI, and physical activity were treated as continuous variables and sex, monthly household income, smoking status, alcohol consumption status, and the presence of hypertension and diabetes mellitus as categorical variables. We confirmed no missing data for continuous covariates and created an additional group for missing data of categorical covariates. Further analysis stratified by sex and age groups (age < 65 years and ≥ 65 years) was also conducted. All tests were based on a two-sided level of significance, and SAS, version 9.4 software was used (SAS Institute, Cary, NC, USA).

## Supplementary Information

Supplementary Information.

## Data Availability

Datasets are available in a funder- mandated (website of the Korea Disease Control and Prevention Agency: https://is.kdca.go.kr/).

## References

[1] Harley CB, Futcher AB, Greider CW (1990). Telomeres shorten during ageing of human fibroblasts. Nature.

[2] Li L, Lejnine S, Makarov V, Langmore JP (1998). In vitro and in vivo reconstitution and stability of vertebrate chromosome ends. Nucleic Acids Res..

[3] Müezzinler A, Zaineddin AK, Brenner H (2013). A systematic review of leukocyte telomere length and age in adults. Ageing Res. Rev..

[4] Valdes AM, Andrew T, Gardner JP, Kimura M, Oelsner E, Cherkas LF (2005). Obesity, cigarette smoking, and telomere length in women. Lancet.

[5] Demissie S, Levy D, Benjamin EJ, Cupples LA, Gardner JP, Herbert A (2006). Insulin resistance, oxidative stress, hypertension, and leukocyte telomere length in men from the Framingham Heart Study. Aging Cell.

[6] Aviv A (2002). Telomeres, sex, reactive oxygen species, and human cardiovascular aging. J. Mol. Med..

[7] Grodstein F, van Oijen M, Irizarry MC, Rosas HD, Hyman BT, Growdon JH, De Vivo I (2008). Shorter telomeres may mark early risk of dementia: Preliminary analysis of 62 participants from the Nurses’ Health Study. PLoS ONE.

[8] Blasco MA (2005). Telomeres and human disease: Ageing, cancer and beyond. Nat. Rev. Genet..

[9] Cawthon RM, Smith KR, O’Brien E, Sivatchenko A, Kerber RA (2003). Association between telomere length in blood and mortality in people aged 60 years or older. Lancet.

[10] Andrew T, Aviv A, Falchi M, Surdulescu GL, Gardner JP, Lu X (2006). Mapping genetic loci that determine leukocyte telomere length in a large sample of unselected female sibling pairs. Am. J. Hum. Genet..

[11] Nettleton JA, Diez-Roux A, Jenny NS, Fitzpatrick AL, Jacobs DR (2008). Dietary patterns, food groups, and telomere length in the Multi-Ethnic Study of Atherosclerosis (MESA). Am. J. Clin. Nutr..

[12] Lee JY, Jun NR, Yoon D, Shin C, Baik I (2015). Association between dietary patterns in the remote past and telomere length. Eur. J. Clin. Nutr..

[13] Shin C, Baik I (2016). Associations between alcohol consumption and leukocyte telomere length modified by a common polymorphism of ALDH2. Alcohol. Clin. Exp. Res..

[14] Chan R, Woo J, Suen E, Leung J, Tang N (2010). Chinese tea consumption is associated with longer telomere length in elderly Chinese men. Br. J. Nutr..

[15] Tucker LA (2017). Caffeine consumption and telomere length in men and women of the National Health and Nutrition Examination Survey (NHANES). Nutr. Metab..

[16] Leung CW, Laraia BA, Needham BL, Rehkopf DH, Adler NE, Lin J (2014). Soda and cell aging: Associations between sugar-sweetened beverage consumption and leukocyte telomere length in healthy adults from the national health and nutrition examination surveys. Am. J. Public Health.

[17] Nonino CB, Caressato Pinhanelli V, Noronha NY, Quinhoneiro DCG, Souza Pinhel MA, De Oliveira BAP (2018). Green tea supplementation promotes leukocyte telomere length elongation in obese women. Nutr. Hosp..

[18] Teramoto M, Muraki I, Yamagishi K, Tamakoshi A, Iso H (2021). Green tea and coffee consumption and all-cause mortality among persons with and without stroke or myocardial infarction. Stroke.

[19] Duggan C, Risques R, Alfano C, Prunkard D, Imayama I, Holte S (2014). Change in peripheral blood leukocyte telomere length and mortality in breast cancer survivors. J. Natl. Cancer Inst..

[20] Goglin SE, Farzaneh-Far R, Epel ES, Lin J, Blackburn EH, Whooley MA (2016). Change in leukocyte telomere length predicts mortality in patients with stable coronary heart disease from the heart and soul study. PLoS ONE.

[21] Cabrera C, Artacho R, Giménez R (2006). Beneficial effects of green tea—A review. J. Am. Coll. Nutr..

[22] McKay DL, Blumberg JB (2002). The role of tea in human health: An update. J. Am. Coll. Nutr..

[23] Gardner EJ, Ruxton CHS, Leeds AR (2007). Black tea—Helpful or harmful? A review of the evidence. Eur. J. Clin. Nutr..

[24] Rice-Evans C (1999). Implications of the mechanisms of action of tea polyphenols as antioxidants in vitro for chemoprevention in humans. Proc. Soc. Exp. Biol. Med..

[25] Bhagwat, S., Haytowitz, D.B. & Holden, J.M. *USDA Database for the Flavonoid Content of Selected Foods, Release 3.1* (2012).

[26] Juśkiewicz J, Zduńczyk Z, Jurgoński A, Brzuzan Ł, Godycka-Kłos I, Zary-Sikorska E (2008). Extract of green tea leaves partially attenuates streptozotocin-induced changes in antioxidant status and gastrointestinal functioning in rats. Nutr. Res..

[27] Donà M, Dell’Aica I, Calabrese F, Benelli R, Morini M, Albini A, Garbisa S (2003). Neutrophil restraint by green tea: Inhibition of inflammation, associated angiogenesis, and pulmonary fibrosis. J. Immunol..

[28] von Zglinicki T (2002). Oxidative stress shortens telomeres. Trends Biochem. Sci..

[29] Oikawa S, Kawanishi S (1999). Site-specific DNA damage at GGG sequence by oxidative stress may accelerate telomere shortening. FEBS Lett..

[30] Saretzki G, Von Zglinicki T (2002). Replicative aging, telomeres, and oxidative stress. Ann. N. Y. Acad. Sci..

[31] Ni J, Guo X, Wang H, Zhou T, Wang X (2018). Differences in the effects of EGCG on chromosomal stability and cell growth between normal and colon cancer cells. Molecules.

[32] Barrett ELB, Richardson DS (2011). Sex differences in telomeres and lifespan. Aging Cell.

[33] Liu JJ, Crous-Bou M, Giovannucci E, De Vivo I (2016). Coffee consumption is positively associated with longer leukocyte telomere length in the Nurses' Health Study. J. Nutr..

[34] El-Terras A, Soliman MM, Alkhedaide A, Attia HF, Alharthy A, Banaja AE (2016). Carbonated soft drinks induce oxidative stress and alter the expression of certain genes in the brains of Wistar rats. Mol. Med. Rep..

[35] Daniali L, Benetos A, Susser E, Kark JD, Labat C, Kimura M (2013). Telomeres shorten at equivalent rates in somatic tissues of adults. Nat. Commun..

[36] Wang CJY, Warner JK, Erdmann N, Lansdorp PM, Harrington L, Dick JE (2005). Dissociation of telomerase activity and telomere length maintenance in primitive human hematopoietic cells. Proc. Natl. Acad. Sci. USA.

[37] Baik I, Shin C (2008). Prospective study of alcohol consumption and metabolic syndrome. Am. J. Clin. Nutr..

[38] Kim Y, Han BG, KoGES group (2017). Cohort profile: The Korean Genome and Epidemiology Study (KoGES) consortium. Int. J. Epidemiol..

[39] Fred Hutchinson Cancer Research Center. *Nutrition Assessment Shared Resources: Caffeine Questionnaire* (Fred Hutchinson Cancer Research Center, Seattle, 2022). https://www.fredhutch.org/en/research/divisions/public-health-sciences-division/research/nutrition-assessment.html#short. Accessed 30 June 2022.

[40] Cawthon RM (2002). Telomere measurement by quantitative PCR. Nucleic Acids Res..

[41] Ainsworth BE, Haskell WL, Whitt MC, Irwin ML, Swartz AM, Strath SJ (2000). Compendium of physical activities: An update of activity codes and MET intensities. Med. Sci. Sports Exerc..

