# Supplementary material for: Associations of green tea, coffee, and soft drink consumption with longitudinal changes in leukocyte telomere length
Source: Sci Rep. 2023 Jan 10;13:492. doi: 10.1038/s41598-022-26186-y (PMC9832020; doi:10.1038/s41598-022-26186-y)
Supplement: Supplementary file 1 — Supplementary Information. [file 41598_2022_26186_MOESM1_ESM.docx]

Supplemental Table S1. Associations of beverage consumption with baseline and follow-up leukocyte telomere length

| Beverage consumption |  | |  | Model^2^ for baseline | Model^2^ for follow-up |
| --- | --- | --- | --- | --- | --- |
| Types | Categories | N^1^ | | Estimate^3^ (95% CI) | Estimate^3^ (95% CI) |
| Green tea | None | 1,505 | | Reference | Reference |
|  | <7 cups/week | 227 | | -0.002 (-0.044, 0.040) | 0.028 (-0.018, 0.074) |
|  | ≥7 cups/week | 220 | | -0.029 (-0.072, 0.013) | 0.049 (0.003, 0.096)^5^ |
| Others^4^ | None | 1,910 | | Reference | Reference |
|  | ≥1 cup/month | 42 | | -0.083 (-0.175, 0.009) | -0.041 (-0.141, 0.059) |
| Brewed coffee | None | 1,563 | | Reference | Reference |
|  | <14 cups/week | 217 | | 0.012 (-0.032, 0.055) | -0.022 (-0.069, 0.025) |
|  | ≥14 cups/week | 172 | | -0.023 (-0.071, 0.025) | -0.034 (-0.086, 0.018) |
| Instant coffee | None | 755 | | Reference | Reference |
|  | <14 cups/week | 518 | | -0.012 (-0.045, 0.022) | 0.010 (-0.027, 0.047) |
|  | ≥14 cups/week | 679 | | 0.002 (-0.031, 0.035) | 0.022 (-0.014, 0.058) |
| Soft drink | None | 1,870 | | Reference | Reference |
|  | ≥1 cup/month | 82 | | -0.057 (-0.124, 0.010) | 0.019 (-0.054, 0.092) |

Abbreviation: CI, confidence interval

^1^ Number of participants

^2^ Model for leukocyte telomere length adjusted for age, sex, monthly household income status, employment status, body mass index, smoking status, alcohol consumption status, physical activity, white blood cell counts, and presence of hypertension or diabetes mellitus.

^3^ Regression coefficient estimate

^4^ Black tea, oolong tea, and other teas

^5^*P*-value < 0.05
